# Occurrence of Heart Failure-Related Clinical Signs in a Japanese Convalescent Rehabilitation Ward: A Cross-Sectional Pilot Study

**DOI:** 10.7759/cureus.116727

**Published:** 2026-09-22

**Authors:** Masato Ando, Daisuke Kimura, Naoki Tomiyama, Kenta Kunoh, Ryuichi Hasegawa

**Affiliations:** 1 Department of Rehabilitation, Ogaki Municipal Hospital, Gifu, JPN; 2 Department of Rehabilitation, Yamada Hospital, Gifu, JPN; 3 Department of Rehabilitation, Faculty of Health Sciences, Naragakuen University, Nara, JPN; 4 Faculty of Care and Rehabilitation, Seijoh University, Tokai, JPN; 5 Graduate School of Life and Health Sciences, Chubu University, Kasugai, JPN

**Keywords:** cardiomegaly, convalescence, cross-sectional studies, heart failure, japanese rehabilitation

## Abstract

Background

Heart failure (HF) is a major contributor to cardiovascular morbidity. It is common among older adults and often characterized by progression, repeated healthcare needs, declines in cardiac and physical function, and reduced quality of life. This pilot study aimed to describe the occurrence of HF-related clinical signs among patients admitted to a Japanese convalescent rehabilitation ward.

Methods

This single-center cross-sectional study included 128 patients without marked cognitive impairment. Rehabilitation professionals assessed seven noninvasive clinical items selected from the Framingham criteria. Based on the presence of a physician diagnosis of HF and the assessed clinical signs, the patients were classified into three groups: the physician-diagnosed HF, sign-positive, and non-sign groups.

Results

Of the 128 patients, 20, 49, and 59 were classified into the physician-diagnosed HF, sign-positive, and non-sign groups, respectively. Cardiomegaly was frequently observed in both the physician-diagnosed HF and sign-positive groups, whereas the other major criteria were rarely identified.

Conclusion

Our findings indicate that HF-related clinical signs may also be present in patients without a formal diagnosis of HF. A substantial proportion of patients without physician-diagnosed HF showed HF-related clinical signs in a convalescent rehabilitation ward. Screening for these signs by rehabilitation professionals may help identify patients who require closer observation and may contribute to safer rehabilitation practice and risk management.

## Introduction

Cardiovascular disease is a leading cause of death worldwide, and heart failure (HF) is a major contributor to cardiovascular morbidity, hospitalization, health care burden, and mortality [1,2]. HF is common among older adults and often follows a gradual and progressive course characterized by repeated hospitalization, stepwise declines in cardiac and physical function, and reduced quality of life [3,4]. The etiology of HF is heterogeneous and includes direct myocardial injury, long-term pressure or volume overload, and hemodynamic deterioration related to arrhythmia or other cardiovascular disorders [3-6]. HF has traditionally been classified according to left ventricular ejection fraction (LVEF) into HF with reduced ejection fraction (LVEF ≤40%), HF with mildly reduced ejection fraction (LVEF 41-49%), and HF with preserved ejection fraction (LVEF ≥50%) [3,4]. However, recent international guidance has simplified LVEF-based phenotyping and emphasized the limitations of rigid numerical cutoffs [5,6]. Symptomatic severity is commonly described using the New York Heart Association (NYHA) functional classification, ranging from class I (no limitation of physical activity) to class IV (symptoms at rest) [3,4]. These pathological conditions may worsen because of aging and lifestyle-related risk factors [2,7], and the burden of HF globally, including that in Japan, has continued to increase [4].

Many patients admitted to convalescent rehabilitation wards in Japan are older adults, and annual survey data from the Japan Convalescent Rehabilitation Ward Association indicate that approximately 70% of patients are aged ≥75 years [8]. Convalescent rehabilitation wards are a distinctive component of the Japanese health care system, providing intensive multidisciplinary rehabilitation after acute care and supporting functional recovery and transition to home or community living [9]. Regarding the diseases treated in these wards, cerebrovascular and musculoskeletal diseases account for most admissions [8]. Cardiovascular disease is clinically relevant in stroke rehabilitation, because stroke and coronary events share many vascular risk factors [10]. In patients undergoing musculoskeletal rehabilitation, HF may limit physical activity and rehabilitation tolerance among older adults [11]. Therefore, even among patients who have not been diagnosed with HF, a proportion of patients admitted to convalescent rehabilitation wards may present with HF-related clinical signs, such as dyspnea, orthopnea, peripheral edema, cardiomegaly, and pleural effusion.

The convalescent phase is an important transition period after acute care, during which patients receive intensive rehabilitation for functional recovery and are discharged home. Simultaneously, in patients with signs of HF, excessive exercise load during rehabilitation may increase the risk of acute decompensation. Recent HF guidelines and consensus definitions emphasize that symptoms and signs are important for clinical suspicion but are insufficient by themselves to establish a diagnosis of HF; objective evidence of cardiac structural or functional abnormalities is also required [3-6]. Therefore, in rehabilitation practice, observable HF-related signs should not be regarded as diagnostic evidence of HF. Rather, their presence should alert rehabilitation professionals to the need for closer patient monitoring, possible adjustment of exercise intensity, and timely communication with physicians and nurses when clinical deterioration is suspected. However, the occurrence of HF-related signs in patients admitted to convalescent rehabilitation wards remains unclear. In this pilot study, we aimed to investigate the presence or absence of a physician diagnosis of HF and the occurrence of HF-related clinical signs among inpatients in a convalescent rehabilitation ward and to obtain preliminary data for risk management during rehabilitation.

## Materials and methods

Participants

This was a single-center, cross-sectional pilot study. Of 425 patients admitted to the convalescent rehabilitation ward of the authors' institution between September 27, 2023, and October 10, 2024, 128 who did not meet the exclusion criteria were included in the analysis (Figure 1). The exclusion criteria were as follows: (1) patients who could not undergo screening using the Hasegawa Dementia Scale-Revised (HDS-R) [12] or who had an HDS-R score of ≤20 points and (2) patients diagnosed with respiratory diseases, such as chronic obstructive pulmonary disease, pulmonary fibrosis, interstitial pneumonia, asthma, and chronic bronchitis (Table 1). Information on participant characteristics, including sex, age, and type of disease-specific rehabilitation fee category, was collected from electronic medical records. LVEF and NYHA functional class were not systematically collected as predefined variables; therefore, patients were not classified according to LVEF-based HF phenotype or symptomatic severity.

**Figure 1:**
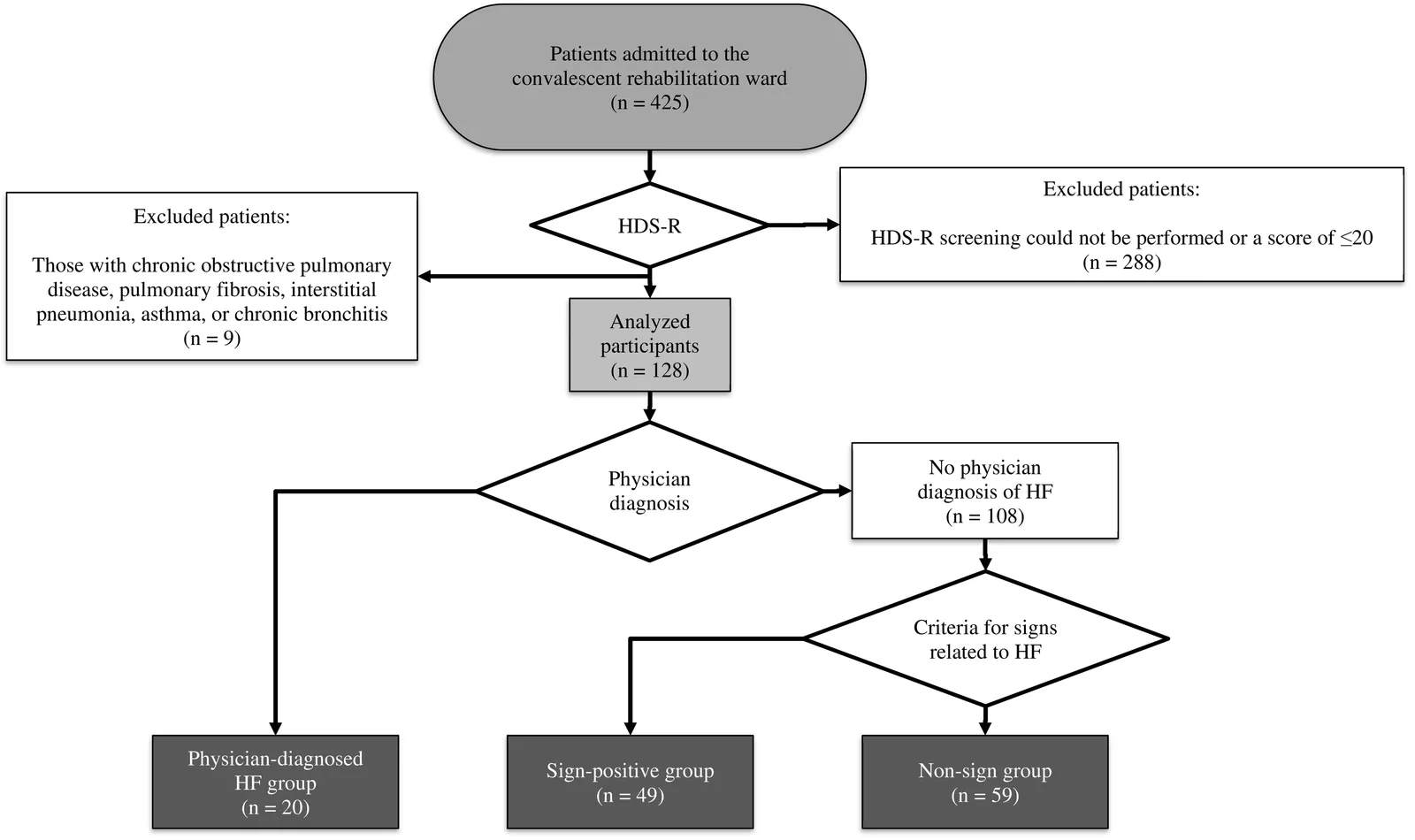
Flowchart of participant selection and classification HF: heart failure; HDS-R: Hasegawa Dementia Scale-Revised

**Table 1:** Inclusion and exclusion criteria HDS-R: Hasegawa Dementia Scale-Revised

Criterion	Details
Inclusion	Admission to the convalescent rehabilitation ward between September 27, 2023, and October 10, 2024
Exclusion	Unable to complete HDS-R screening or an HDS-R score of 20 points or less
	Diagnosis of respiratory diseases, including chronic obstructive pulmonary disease, pulmonary fibrosis, interstitial pneumonia, asthma, or chronic bronchitis

Assessment of HF-related signs

Clinical signs related to HF were evaluated using selected components of the Framingham criteria [13], which consist of clinical features related to HF categorized as major and minor criteria. The major criteria include paroxysmal nocturnal dyspnea or orthopnea, neck vein distention, rales, cardiomegaly on chest radiography, acute pulmonary edema, S3 gallop, increased central venous pressure, prolonged circulation time, and hepatojugular reflux. The minor criteria include ankle edema, nocturnal cough, dyspnea on exertion, hepatomegaly, pleural effusion, decreased vital capacity, tachycardia, and weight loss.

As several of these criteria require procedures or diagnostic maneuvers that are generally performed by physicians, their application in routine independent assessments by physical or occupational therapists is not necessarily feasible. Therefore, in this study, a modified set of seven noninvasive items that physical and occupational therapists can generally identify during routine rehabilitation practice was used: three major criteria (paroxysmal nocturnal dyspnea or orthopnea, neck vein distention, and cardiomegaly) and four minor criteria (ankle edema, nocturnal cough, dyspnea on exertion, and pleural effusion). Specifically, patients without a physician diagnosis of HF were categorized as showing HF-related signs in the presence of at least one item corresponding to a major criterion or at least two items corresponding to minor criteria, based on previous research and clinical practice guidelines for HF [3,13,14]. These patients were defined as the sign-positive group in this study (Table 2). This classification was intended to identify clinically observable signs that may require caution during rehabilitation and do not constitute a medical diagnosis of HF or reduced cardiac function. Patients with HF were defined as those with a physician-documented diagnosis of HF in their medical records. Acute and chronic HF were not separately classified.

**Table 2:** Classification criteria for patients with signs related to heart failure Modified from Velagaleti et al. [14].

		Minor criteria		
		2 or more	1	None
Major criteria	1 or more	Sign-positive group	Sign-positive group	Sign-positive group
	None	Sign-positive group	Non-sign group	Non-sign group

Paroxysmal Nocturnal Dyspnea or Orthopnea

Paroxysmal nocturnal dyspnea and orthopnea were assessed using separate self-reported questionnaire items. If a participant answered "yes" to either question (Table 3, Items A and B), they were recorded as having paroxysmal nocturnal dyspnea or orthopnea.

**Table 3:** Questionnaire This questionnaire was developed by the authors with reference to the Heart Failure Self-Check Sheet of the Japanese Circulation Society [15].

Question	Response
(A) Do you ever feel short of breath while sleeping or lying down?	□ Yes (go to question B)
	□ No (go to question C)
(B) When this occurs, does sitting up or getting out of bed relieve the symptom?	□ Yes
	□ No
(C) Do you wake up at night because of coughing?	□ Yes
	□ No
(D) Do you experience shortness of breath when walking or after climbing 12 to 14 stairs?	□ Yes
	□ No
(E) Have you ever been diagnosed with heart disease (left ventricular hypertrophy, myocardial infarction, valvular disease, arrhythmia, or cardiomyopathy)?	□ Yes
	□ No
(F) Are you currently receiving treatment for lifestyle-related diseases (hypertension, diabetes mellitus, or dyslipidemia)?	□ Yes
	□ No
(G) Do you feel short of breath when bending forward, such as when putting on shoes or socks?	□ Yes
	□ No
(H) During this hospitalization, has anyone told you that your hands or feet are cold?	□ Yes
	□ No

Neck Vein Distention

Neck vein distention was assessed with the patient's upper body elevated to 45º by using the bed. When the two occupational therapists observed neck vein distention, the vertical distance from the sternal angle to the highest point of the internal jugular venous pulsation on the cranial side was measured. A distance of ≥3 cm was recorded as indicative of neck vein distention [3,4].

Cardiomegaly

Cardiomegaly was evaluated using the cardiothoracic ratio (CTR) calculated from chest radiographs, because all patients admitted to the convalescent rehabilitation ward of the study hospital underwent chest radiography on admission. Two occupational therapists reviewed the chest radiographs stored in electronic medical records and calculated the CTR. A CTR of ≥50% was treated as indicative of cardiomegaly [16].

Ankle Edema

To evaluate edema, a pitting test is often performed by pressing the distal one-third of the anterior tibial area bilaterally or the dorsum of the foot with the thumb for approximately 10 seconds [17]. In this study, the dorsum of the foot was used. Three physical or occupational therapists performed the pitting test and observed whether a depression of ≥4 mm remained. Edema was recorded as present only when all three therapists agreed.

Nocturnal Cough

Nocturnal cough was initially evaluated using a self-reported questionnaire. If a participant reported a cough on the questionnaire, the evaluator verbally confirmed whether the cough was wet at the time the questionnaire was collected. If this was confirmed, the participant was recorded as having a nocturnal cough (Table 3, Item C).

Dyspnea on Exertion

Dyspnea on exertion was assessed using a questionnaire that asked participants about dyspnea during exertional activities, such as walking or climbing stairs. If the participant answered "yes," they were recorded as having dyspnea on exertion (Table 3, Item D).

Pleural Effusion

Pleural effusion was assessed using radiological interpretations documented by physicians in medical records. If no physician interpretation was available, two occupational therapists reviewed the chest radiographs obtained on admission to determine whether fluid accumulation was present in the bilateral pleural cavities. Pleural effusion was recorded as present only when both therapists agreed on its presence. In contrast, pleural effusion was recorded as absent when the therapists' findings did not agree, when obvious pleural effusion could not be confirmed, or when abnormal blood test results due to renal or hepatic dysfunction suggested a possible non-cardiac cause. Because pleural effusion has multiple etiologies and often requires differential diagnostic information, it was interpreted cautiously as a clinical sign rather than as evidence of cardiac dysfunction [18].

Statistical analysis

The patients were divided into three groups: the physician-diagnosed HF, sign-positive, and non-sign groups. Descriptive statistics were calculated for each item according to group. Sex and disease categories were compared using Fisher's exact test, followed by Bonferroni multiple comparisons to clarify intergroup differences. Age was compared using the Kruskal-Wallis test, followed by Bonferroni multiple comparisons.

Because the seven HF-related clinical signs used in this study (paroxysmal nocturnal dyspnea or orthopnea, neck vein distention, cardiomegaly, ankle edema, nocturnal cough, dyspnea on exertion, and pleural effusion) were used as criteria for group classification, they were not subjected to comparative hypothesis testing as independent outcomes. Instead, their occurrence rates were exploratorily described to confirm their clinical patterns and feasibility for risk monitoring.

Statistical analyses were performed using EZR (Easy R) version 1.68 (Saitama Medical Center, Jichi Medical University, Saitama, Japan). The statistical significance level was set at p < .05.

Ethics approval

The study outline and handling of personal information were explained to participants both orally and in writing. After patients provided written informed consent to participate, assessments and questionnaires were administered. In addition, information regarding the use of electronic medical records was posted on the hospital bulletin board, and the patients and their family members were given the opportunity to opt out.

This study was conducted after approval was obtained from the Chubu University Ethics Review Committee and permission was granted by the university president (approval number: 20230053).

## Results

Of the 128 participants included in the analysis, 20 were classified into the physician-diagnosed HF group. The remaining 108 patients without a physician diagnosis of HF were further classified according to the presence or absence of HF-related signs defined in this study. Accordingly, 49 and 59 patients were classified into the sign-positive and non-sign groups, respectively (Figure 1).

Regarding participant characteristics by group, no significant differences in sex were observed among the three groups. Age differed significantly among the groups (p < .001): The mean ages were 79 ± 10, 75 ± 11, and 63 ± 16 years in the physician-diagnosed HF, sign-positive, and non-sign groups, respectively. Post hoc comparisons showed that the non-sign group was significantly younger than both the physician-diagnosed HF and sign-positive groups (Table 4).

**Table 4:** Participant characteristics by group Values are presented as n (%) or mean ± SD. Overall, p-values are shown. a: Fisher's exact test was used for the sex and disease categories; b: the Kruskal-Wallis test was used for age. Post hoc test: Bonferroni-adjusted multiple comparisons. *p < .05. HF: heart failure; NS: not significant; SD: standard deviation

	A: Physician-diagnosed HF group n = 20	B: Sign-positive group n = 49	C: Non-sign group n = 59	P-value	Post hoc test
Sex^a^					
Men	7 (35%)	20 (41%)	32 (54%)	0.144	NS
Women	13 (65%)	29 (59%)	27 (46%)		
Age, years^b^	79 ± 10	75 ± 11	63 ± 16	< .001*	A > C, B > C
Disease category^a^					
Cerebrovascular	7 (35%)	22 (45%)	39 (66%)	< .001*	NS
Musculoskeletal	11 (55%)	27 (55%)	19 (32%)		
Disuse syndrome	2 (10%)	0 (0%)	1 (2%)		

Regarding disease-specific rehabilitation fee categories, the total sample included 68, 57, and 3 patients with cerebrovascular disease, musculoskeletal disease, and disuse syndrome, respectively. The distribution of disease categories differed significantly among the three groups (p < .001). However, post hoc comparisons showed no significant differences in any pairwise combination of the groups (Table 4).

Descriptive examinations of the seven HF-related signs used in this study showed distinct clinical patterns (Table 5). Cardiomegaly was frequently observed in the physician-diagnosed HF group (75%) and sign-positive group (92%). The occurrence rates of the other two major criteria, besides cardiomegaly, were low (≤5%) in all groups. In contrast, the occurrence rates of the four minor criteria were ≤10% in the non-sign group, and all four items were observed in at least 15% of the patients in the physician-diagnosed HF group. Furthermore, ankle edema was observed in 40% of the patients in that group. In the sign-positive group, the occurrence rates of the four minor criteria tended to fall between those in the physician-diagnosed HF and non-sign groups (Table 5).

**Table 5:** Occurrence of signs related to heart failure by group Values are presented as n (%). Percentages were calculated based on the number of patients in each group. Because these signs were used as criteria for group classification, no comparative hypothesis testing was conducted. HF: heart failure

	Signs related to heart failure	Status	Overall	A: Physician-diagnosed HF group	B: Sign-positive group	C: Non-sign group
			N = 128	n = 20	n = 49	n = 59
Major criteria	Paroxysmal nocturnal dyspnea or orthopnea	Present	2 (2%)	1 (5%)	1 (2%)	0 (0%)
		Absent	126 (98%)	19 (95%)	48 (98%)	59 (100%)
	Neck vein distention	Present	0 (0%)	0 (0%)	0 (0%)	0 (0%)
		Absent	128 (100%)	20 (100%)	49 (100%)	59 (100%)
	Cardiomegaly	Present	60 (47%)	15 (75%)	45 (92%)	0 (0%)
		Absent	68 (53%)	5 (25%)	4 (8%)	59 (100%)
Minor criteria	Ankle edema	Present	26 (20%)	8 (40%)	12 (24%)	6 (10%)
		Absent	102 (80%)	12 (60%)	37 (76%)	53 (90%)
	Nocturnal cough	Present	8 (6%)	3 (15%)	5 (10%)	0 (0%)
		Absent	120 (94%)	17 (85%)	44 (90%)	59 (100%)
	Dyspnea on exertion	Present	22 (17%)	7 (35%)	11 (22%)	4 (7%)
		Absent	106 (83%)	13 (65%)	38 (78%)	55 (93%)
	Pleural effusion	Present	23 (18%)	6 (30%)	12 (24%)	5 (8%)
		Absent	105 (82%)	14 (70%)	37 (76%)	54 (92%)

## Discussion

We investigated the occurrence of HF-related clinical signs among inpatients in a convalescent rehabilitation ward in Japan. In addition to patients with physician-diagnosed HF, a substantial number of patients without physician-diagnosed HF met the positive sign criteria used in this study. These findings suggest that relying solely on an existing diagnosis of HF may be insufficient to identify patients who require careful monitoring during rehabilitation.

No significant differences in sex were observed between the groups. Sex differences in cardiovascular disease and HF have been described in epidemiological studies; however, the relationship between sex and HF is influenced by age, HF phenotype, and cardiovascular risk factors [7,19-21]. In this older convalescent rehabilitation cohort, such factors may have attenuated sex-related differences in the occurrence of HF-related signs.

Patient age was significantly lower in the non-sign group than in the physician-diagnosed and sign-positive HF groups. Aging is associated with structural and functional cardiovascular changes, including increased ventricular and vascular stiffness, impaired reserve capacity, and a greater burden of comorbid cardiovascular conditions [7,21,22]. Therefore, the lower patient age in the non-sign group may indicate that these patients had not yet reached the stage at which clinically observable signs, such as fluid retention or exertional intolerance, were apparent.

Regarding disease category, 68 and 57 patients had cerebrovascular and musculoskeletal diseases, respectively, whereas only three patients had disuse syndrome. Although the distribution of disease categories differed significantly between the groups, this finding may have been affected by the extremely small number of patients with disuse syndrome and the resulting statistical instability. Future multicenter studies with larger and more balanced samples are needed to determine whether the occurrence of HF-related signs differs according to rehabilitation diagnosis.

The main clinical implication of this study is that some patients without a formal HF diagnosis may present with clinically observable HF-related signs in convalescent rehabilitation wards. Cardiomegaly was frequently observed in the sign-positive group, and signs such as ankle edema, pleural effusion, and dyspnea upon exertion were also present in some patients. Although these findings can occur in association with HF or reduced cardiac function, this study did not systematically collect objective cardiac measures, including echocardiographic parameters such as LVEF or biomarkers such as brain natriuretic peptide (BNP) and N-terminal pro-BNP (NT-proBNP). Therefore, patients could not be classified according to LVEF-based HF phenotypes. The purpose of this study was not to diagnose or phenotype HF but to describe readily observable HF-related clinical signs in a convalescent rehabilitation setting. Current guidelines emphasize that symptoms and signs alone are insufficient to establish a diagnosis of HF and that objective evidence of cardiac structural or functional abnormalities is required [3-6]. Therefore, these findings cannot be interpreted as evidence of progressive ventricular dysfunction or as a medical diagnosis of HF. Nevertheless, identifying these observable parameters may help physical and occupational therapists monitor patient status over time and share information with physicians and nurses to support safe rehabilitation.

Among the signs assessed, cardiomegaly can be identified on routine chest radiography and may be confirmed even in patients who report few subjective symptoms. However, CTR is an indirect radiographic indicator and may be influenced by factors other than cardiac function, such as lung volume, body position, and thoracic configuration [16]. Therefore, in the absence of echocardiographic or biomarker confirmation, an elevated CTR should not be interpreted as definitive evidence of cardiac dysfunction. Instead, it should be considered a clinical warning sign that warrants cautious exercise prescriptions and interdisciplinary communication during rehabilitation.

A descriptive examination of these four minor criteria also warrants further clinical attention. Ankle edema may reflect venous congestion and fluid retention, and a pitting test is a feasible bedside method for evaluating peripheral edema [17]. Dyspnea on exertion directly affects daily activities and rehabilitation tolerance and is associated with reduced quality of life in patients with HF [23]. Pleural effusion may occur in HF but has multiple possible causes, and biomarkers or additional diagnostic information may be needed for etiological interpretation [18]. However, because these signs are nonspecific and may be caused by renal dysfunction, respiratory disease, venous disease, or other conditions, they should be interpreted in the context of each patient's overall clinical background.

Clinical symptoms caused by cardiac dysfunction are often mild or not perceived in the early stages, and patients may not experience difficulty in daily life when functional decline is minimal [22,24]. As symptoms progress, chronic volume or pressure overload may increase ventricular wall stress and contribute to myocardial remodeling characterized by cellular stretching, hypertrophy, and fibrosis [24]. In the early stage, cardiac output may be maintained through compensatory mechanisms, such as the Frank-Starling mechanism. When compensation is insufficient, decreased cardiac output and elevated venous pressure may occur, and clinical symptoms, such as edema and dyspnea, may become apparent [3,21,24]. Thus, progressive cardiac dysfunction may affect daily life, reduce independence, and increase the risk of rehospitalization. Therefore, systematic screening of physically observable and clinically accessible signs may be useful for bedside risk management in convalescent rehabilitation.

Because this study was cross-sectional, it could not clarify the order in which HF-related signs appear or the process by which cardiac function declines. However, the finding that a substantial number of patients without a physician diagnosis of HF met the criteria for HF-related signs suggests the clinical value of routine screening. Therefore, physical and occupational therapists should not use these signs as surrogate diagnostic tools. Instead, they should be used as practical clinical cues for risk monitoring, exercise load adjustment, and timely interdisciplinary communication.

Limitations and future directions

This study had some limitations. First, because the participants were recruited from a single facility, the characteristics of the study population may have been influenced by the hospital's specific features, leading to a possible bias in the proportion of patients with cerebrovascular or musculoskeletal diseases and age distribution. Therefore, future studies should examine the trends among patients admitted to other convalescent rehabilitation wards.

Second, we extracted and used seven items from the Framingham criteria and HF clinical practice guidelines that were considered identifiable by physical and occupational therapists in routine clinical practice. However, the diagnostic validity and prognostic accuracy of this modified 7-item classification method have not been verified against the full diagnostic criteria, echocardiographic parameters such as LVEF, or cardiac biomarkers. Because LVEF was not systematically collected, patients could not be classified according to LVEF-based HF phenotypes. Therefore, patients classified as sign-positive in this study represent a population of patients meeting the criteria established for this study and should not be interpreted as having a medical diagnosis of HF, a specific HF phenotype, or reduced cardiac function. Future prospective studies should include patients with cognitive impairment, where feasible, and examine HF-related signs by combining imaging findings such as CTR, physical signs, biomarkers such as BNP or NT-proBNP, and physician diagnostic information.

Third, because the HF-related signs used in this study were also used for group classification, there is inherent circularity in interpreting the differences in item-specific occurrence rates as independent between-group differences. Therefore, the present study limited the analysis of these items to a descriptive exploration. Future studies should examine the clinical significance of individual signs, such as ankle edema, nocturnal cough, dyspnea on exertion, and pleural effusion, by using independent reference standards and rigorous differential diagnosis protocols.

Finally, some patients with cognitive impairment who were excluded based on HDS-R may also have met the criteria for HF-related signs. The exclusion of these patients may have led to an underestimation of the occurrence of HF-related signs in the broader convalescent rehabilitation population. Future prospective studies should include patients with cognitive impairment, where feasible, and examine HF-related signs by combining imaging findings such as CTR, physical signs, biomarkers such as BNP or NT-proBNP, and physician diagnostic information.

## Conclusions

This study demonstrated that HF-related clinical signs frequently occur among inpatients undergoing convalescent rehabilitation but lacking a formal diagnosis of HF. Although these noninvasive physical signs cannot replace an objective medical diagnosis, their presence highlights potential cardiovascular risks during active physical recovery.

Systematic screening for these accessible clinical cues enables physical and occupational therapists to adjust exercise loads and improve interdisciplinary communication. Ultimately, incorporating such routine monitoring into clinical practice enhances patient safety and overall risk management during the convalescent phase.
